# “Our program manager is a woman for the first time”: perceptions of health managers on what workplace policies and practices exist to advance women’s career progression in the health sector in Kenya

**DOI:** 10.1186/s12939-024-02235-y

**Published:** 2024-07-30

**Authors:** Sally Atieno Odunga, Henry Owoko Odero, Jackline Syonguvi, Michelle Mbuthia, Sonja Tanaka, Sylvia Kiwuwa-Muyingo, Damazo T. Kadengye

**Affiliations:** 1 African Population and Health Research Center, Nairobi, Kenya; 2 Global Health, 50/50, Cambridge, UK; 3 Department of Economics and Statistics, Kabale University, Kabale, Uganda

**Keywords:** Workplace policies, Practices, Women in leadership, Career progression, Health sector, Kenya

## Abstract

**Background:**

Existing evidence suggests that organisation-level policies are important in enabling gender equality and equity in the workplace. However, there is little research exploring the knowledge of health sector employees on whether policies and practices to advance women’s career progression exist in their organisations. In this qualitative study, we explored the knowledge and perspectives of health managers on which of their organisations' workplace policies and practices contribute to the career advancement of women and their knowledge of how such policies and practices are implemented and monitored.

**Methods:**

We employed a purposive sampling method to select the study participants. The study adopted qualitative approaches to gain nuanced insights from the 21 in-depth interviews and key informant interviews that we conducted with health managers working in public and private health sector organisations. We conducted a thematic analysis to extract emerging themes relevant to advancing women’s career progression in Kenya’s health sector.

**Results:**

During the interviews, only a few managers cited the policies and practices that contribute to women’s career advancement. Policies and practices relating to promotion and flexible work schedules were mentioned most often by these managers as key to advancing women's career progression. For instance, flexible work schedules were thought to enable women to pursue further education which led to promotion. Some female managers felt that women were promoted to leadership positions only when running women-focused programs. There was little mention of capacity-building policies like training and mentorship. The health managers reported how policies and practices are implemented and monitored in general, however, they did not state how this is done for specific policies and practices. For the private sector, the health managers stated that implementation and monitoring of these policies and practices is conducted at the institutional level while for the public sector, this is done at the national or county level.

**Conclusions:**

We call upon health-sector organisations in Kenya to offer continuous policy sensitisation sessions to their staff and be deliberate in having supportive policies and other pragmatic interventions beyond policies such as training and mentorship that can enable women’s career progression.

## Introduction

Globally, the health sector workforce has a greater representation of women compared to men [1]. According to the 2019 report by the World Health Organization (WHO), Women in Global Health and Global Health Workforce Network on gender and equity analysis of the global health and social workforce, women constitute 70% of the global health workforce and comprise half of the employed individuals in the health sector in low- and middle-income countries (LMICs) [2]. While women comprise a large proportion of the health workforce in LMICs, they are mostly concentrated in lower-paying, lower-status roles such as nursing and midwifery, or are often engaged in unpaid community health volunteer work, with merely 25% of senior leadership positions held by women [1–3]. A review conducted by Newman [4] in Africa showed that gender disparities and inequalities within the health sector persist due to prevailing employment systems with a significant and growing wage gap between men and women for equivalent work [4, 5].

The health sector in Kenya is made up of a wide variety of public and private healthcare institutions, organisations, and facilities. Women make up 58% of the health care workers overall. However, apart from nurses who are 70% females, other health professional careers are predominantly males [6]. Article 27 of Kenya’s Constitution emphasizes gender equality and non-discrimination; it addresses the right to equality and freedom from discrimination. While it does not specifically reference the health sector, it does lay the groundwork for fostering gender equality in all spheres of life including participation in leadership roles [7].

In Kenya, women working in the health sector reported that some organisational factors that barred them from career advancement included; limited job flexibility to enable them to balance family and work demands, lack of capacity-building programs, lack of supportive supervisors, and discrimination at the workplace [3, 8, 9]. Such organisational barriers have been attributed to the lack of institutional policies, structures and laws focusing on gender equality, sex discrimination, sexual harassment, equal pay and social protection [2]. This is because, for many organisations, there is a lack of attention on the impact of discrimination and inequalities that women face in the workplace [4]. For institutions that have gender transformative policies, there is still much that needs to be done in terms of monitoring and implementation of the policies to ensure there is gender equality [10]. According to the United Nations (2019) enabling environment guidelines, organisations should offer flexible working arrangements, family-friendly policies that enable employees to have a balance between personal, family, and professional commitments, and common standards of conduct to ensure a safe, discrimination-free and supportive workplace. These common standards encompass guiding principles and practices that address harassment in all its forms, misconduct and abuse of power [11].

While there is a significant number of studies focusing on enablers and barriers to women’s career progression in the health sector in Kenya [1, 3, 8, 9, 12], studies focusing on what workplace policies contribute to women’s career advancement are limited. Yet existing evidence shows that policies are essential to advancing gender equality at work [2]. Understanding what workforce policies contribute to women’s career advancement in the health sector through the perspectives of male and female health managers can enable institutions and governments to develop and implement appropriate policies addressing discrimination and inequalities in the workplace.

In this study, we examine the knowledge of health managers in Kenya on their organisations’ workplace policies and practices contributing to the career advancement of women. We also examine their knowledge and perspectives on which of their organisations' workplace policies and practices contribute to the career progression of women and their knowledge of how such policies and practices are implemented and monitored.

## Methods

### Study design

This was a formative qualitative study. We interviewed male and female senior and mid-level health professionals from different health organisations including hospitals, NGOs, and labor organisations in the public and private sectors in Kenya. In this study, the senior health professionals comprised of organisational heads/administrators and human resources managers. On the other hand, mid-level officers were heads of departments, programs and units within the organisation. We used In-Depth Interviews (IDI) and Key Informant Interviews (KII), to explore complex and nuanced experiences and perceptions of individual health managers working in Kenya on which of their organisations' workplace policies and practices contribute to the career advancement of women. Data collection took place between the months of November and December 2022.

### Study population and sampling

We employed a purposive sampling approach to select study participants. Purposive sampling allowed for the selection of participants who were most relevant to the study research questions, and whose experiences and perspectives were expected to provide valuable insights into what organisation-level policies and practices contribute to the career advancement of women in Kenya’s health sector and how such policies and practices are implemented and monitored. Participants were chosen from eight public and private health sector organisations. These eight organisations were purposefully selected to be representatives of different sub-sectors of the health sector including health service delivery organisations, research institutes, and professional bodies in Kenya within the public and private health sector. We interviewed eight men and thirteen women to get perspectives from both male and female participants. Within the eight organisations, at least two participants were selected for either IDIs or KIIs. Interviews were only conducted with participants who were willing, available, and consented to be interviewed. A total of 21 interviews were conducted— seven KIIs with a chief executive officer (CEO), human resource managers (HRs), and administrators and 14 IDIs with mid-level managers. The majority of the participants in the KIIs were male while the majority of participants in the IDIs were female. In addition, more than half of the participants were from the private sector. While we reached out to the same number of participants in the public and private sectors, the bureaucratic process in the public sector could have affected the number of participants from this sector. The selection of participants was based on their roles within the organisation, with a particular focus on those who held leadership positions or had extensive experience working in the health sector.

### Data collection

A team of six trained research assistants conducted the interviews. Consent to participate in the study and record the interviews was sought from participants before the interviews. Study participants signed an informed consent form after agreeing to participate in the interviews. The moderator posed the questions while the note-taker documented the interview in writing and on audio. To enhance participants' comfort and ability to express themselves, the interviews were conducted in the language preferred by the participant (either English or Kiswahili), and later transcribed and translated as necessary. Key informant interviews were conducted with selected participants who were identified as having a high level of knowledge and experience within their organisation. Interviews were conducted face-to-face, either at the participant’s workplace or at a secure location of their choice, which was important to create a comfortable and relaxed environment for participants to express their experiences and views. Finally, all interviews were audio-recorded and transcribed verbatim, ensuring the accuracy and completeness of the data collected.

### Data analysis

Data analysis involved a rigorous and systematic examination of the data to identify themes and patterns related to the research questions. Thematic analysis was chosen as the analytical approach since it allows for a systematic and transparent analysis of the data, ensuring that the findings are grounded in the data collected and accurately reflect participants' experiences and perspectives [13]. Transcribed interviews were read and re-read to gain an in-depth understanding of the data, and initial codes were generated to capture meaningful concepts and ideas. Codes were then reviewed, refined, and grouped into themes to identify broader patterns and trends. Themes were then organized into categories and sub-categories. This helped to develop a comprehensive understanding of the data and provided a structured way to report the findings.

## Results

In this section, we first highlight the profiles of the participants interviewed including their sex, education level, title/position, and the type of organisation that they work for. We then, present the broad themes from the interviews which are: 1) knowledge of workplace policies and practices promoting women’s career progression, 2) perspectives on health sector workplace policies and practices promoting women’s career advancement, and 3) implementation and monitoring of health sector workplace policies and practices.

### Profile of participants

We conducted a total of 21 interviews: 7 KIIs with a CEO, HRs, and administrators and 14 IDIs with mid-level managers as shown in Table 1. The majority of the participants in the KIIs were male while the majority of participants in the IDIs were female. Only IDI participants were asked about their educational qualifications given that we were interested in their personal journey to career advancement as opposed to KIIs, which focused on the organisation. The highest education qualification among the IDI participants was a master’s degree. The majority of the study participants worked in the private sector.

**Table 1:** Respondents distribution by type of interview

**Characteristics**	**Type of interview**	
	KIIs (*n*=7)	IDIs (*n*=14)
**Sex**		
Male	6	2
Female	1	12
**Education**		
Diploma	-	4
Bachelor’s degree	-	8
Master’s degree	-	2
**Title**		
CEO	1	-
HR and administrators	6	-
Mid-level managers	-	14
**Type of organisation**		
Public	2	3
Private	5	11

The summary of the findings by themes, type of organisation and positions of health managers is presented in Table 2

**Table 2:** Summary of findings by themes, type of organisations and positions of health managers

Themes	Policies and practices/sub-themes	Type of organisations			
		**Private**		**Public**	
		**Senior managers**	**Middle-level managers**	**Senior managers**	**Middle-level managers**
**Knowledge of workplace policies and practices policies within their institutions that promote women's career advancement**	Awareness of policies	All of them were aware of these policies and practices	Few of them were aware of these policies and practices. A common reason was that they had not read the policies provided to them	The majority of them were aware of these policies and practices. Those not aware attributed this to policies being held at the county and national level by the human resource department	Few of them were aware of these policies and practices. The common reason was that there was limited dissemination of these policies to them
**Perspectives on what health sector workplace policies and practices promote women’s career advancement in their institutions** *Policies and practices relating to promotion and flexible work schedules were thought to contribute to career advancement. On the other hand, those related to hiring, equal pay, sexual harassment, parental leave, and reproductive rights did not distinctively contribute to career progression but rather hiring and retention of women which is also key for career progression*	Promotion	Women are often prioritized for promotion when the project involves women and girls as opposed to when it does not involve this group	The presence of non-discriminatory promotion policies has enabled some institutions to have a program manager who is a woman for the first time	Gender equity is sometimes considered during promotion to enable more women to be promoted. This is in addition to possessing the required qualifications	Gender balance is sometimes considered during the promotion in addition to skillset, experience, and education qualifications
	Flexible work schedules	Provision of flexible (“flexi”) hours for nursing working women. This enables them to continue working while also taking care of their families	Women who are enrolled in school are allowed to leave work early to pursue their studies and after completion of these studies they advance in their careers	Flexible work schedule was not mentioned as one of the practices and policies promoting women’s career advancement	Flexible work schedule was not mentioned as one of the practices and policies promoting women’s career advancement
	Hiring	Hiring was not mentioned as one of the practices and policies promoting women’s career advancement by these managers	Gender equality is observed during the hiring process in addition to qualifications. This ensures that both men and women have equal chances during hiring	Hiring is sometimes based only on one’s qualifications and competence. However, other times, hiring follows the Constitution's two-thirds gender rule that proposes gender balance during any government appointments	Hiring is sometimes guided by ensuring that there is a gender balance. This contributes to more women being employed
	Equal pay	All employees receive equal pay and benefits. Pay is not determined by gender but rather by the competence and organisations budget	No information on equal pay	Pay is based on education qualification. There is no discrimination in pay based on gender	There is a lack of knowledge about how much individuals are paid within the institution. The assumption is that pay is the same for everyone and is based on educational qualification, experience, and performance
	Sexual harassment	There is a sexual harassment policy for the majority of institutions. However, some do not have this policy because they are implementing an old policy that did not incorporate that	There is a sexual harassment policy for the majority of institutions. This guides in respectfully addressing colleagues	There is a sexual harassment policy for the institutions	There is a sexual harassment policy for the institutions
	Parental leave	Availability of paid maternity leave for 30 days or as per the organization’s guidelines. In addition, paternity leave for about 7 or 10 days	Availability of paid maternity leave and paternity leave but duration is not determined. In some institutions, policies on these leaves are unavailable	Availability of paid maternity leave for three months in accordance with the Ministry of Health guidelines. There are paternity leave days but the duration is not determined	Availability of paid maternity leave for three months
	Reproductive rights	Availability of flexible hours for breastfeeding mothers to enable them to leave work early	Provision of health insurance policies that cover reproductive health needs	Availability of dedicated crèche facilities for the staff’s young children with a caregiver. Working women can bring their children to work after the maternity leave and leave at the facilities. They can then occasionally check on them and breastfeed them	Availability of breastfeeding rooms that enable nursing working mothers to express and store milk for their babies
**Implementation and monitoring of policies**	Who implements the policies?	Policies and practices are implemented at the institution level. The management of the institution has the role of implementing and monitoring the policies	Policies and practices are monitored and implemented at the institutional level. The leadership of the institution has the role of implementing and monitoring the policies	Policies and practices are implemented and monitored at the county level by the Department of Health (for devolved public sector organisations such as hospitals) or at the national level by the Ministry of Health for non-devolved public sector organisations such as medical councils The management at the county or national level has the role of implementing and monitoring the policies	Implementation and monitoring of the policies is carried out mainly by the Human Resources department located at the county level. For some, this role is bestowed upon a Gender committee. On the contrary, others lack a formal structure to conduct the implementation
	How are the policies implemented and monitored?	Leadership conducts periodic dissemination of the policies as a way of keeping their staff informed about the policies in place	Appending signatures on the policies and practices to hold employees accountable. Copies of the policies and practices are also issued to them for reference. In some institutions, there are also regular checks conducted to ensure that staff adhere to the policies in place and are updated on any changes that have happened in the respective policies. Failure to adhere to the policies can lead to loss of jobs	Lack of information on how the policies are implemented and monitored since this is done at the county or national level	The gender committee benchmarks with other “model” organisations to find out what policies they have and how they implement them. The committee then implements similar policies and practices

### Knowledge of workplace policies and practices promoting women’s career progression

The majority of the senior managers in both the public and private sectors mentioned that they were aware of the workplace policies and practices promoting women’s career advancement. The few senior managers unaware of these policies were from the public sector and attributed this to limited exposure to the policies since HRs located at the county and national levels are the custodians of the documents. Unlike the senior managers, only a few of the middle-level health managers in the two sectors reported that they were aware of these policies. The middle-level managers in the public sector noted that their lack of awareness was also a result of limited exposure and dissemination of the policies to them. On the other hand, some of those in the private sector did not know the actual content of the policies as they had not read them as indicated in the excerpt below.

*“I won’t speak much about this because to be honest I have not been able to go through all the policies but am in the process. Allow me to pass that. If I knew you would come today, I would have read all the policies”. IDI_middle-level manager_Female_Private*

### Perspectives on health sector workplace policies and practices promoting women’s career advancement

The health managers mentioned that policies and practices that exist within their organisations to advance gender equality, diversity, and inclusion are those related to mainly seven sub-themes which are hiring, promotion, equal pay, sexual harassment, parental leave, reproductive rights, and flexible working schedules.

*“The policy to ensure equal pay and benefits is there. Policies relating to reproductive health rights for employees where you get your thirty (30) days maternity paid leave are embedded in HR manual. Currently, we have a paternity leave for men and new parents”- KII_senior-level manager_Female_Private*

Policies and practices relating to promotion and flexible work schedules were thought by the health managers to advance women's career advancement. The perspectives of the managers on these two sub-themes based on their sex and sector are described below.

#### a) Promotion

Some female middle-level managers in the private sector reported that they have always had program managers who were men in the past. However, felt that because of the presence of organisations’ policies and practices within their institution that are non-discriminatory and encourage women’s career progression, they have a program manager who is a woman for the first time. This woman rose from being an intern in the institution to a program manager as illustrated in the excerpt.

*“So let me see- well because of non-discriminatory policies, our program manager is a woman for the first time. It has always been men who have always been program managers in the past. She has worked here as an intern progressed as a program officer, progressed as coordination and programs and now as a programs manager and it is through the organisation’s policies that she could be able to access these positions without any barriers and without any limitations. Even now that she has taken the program manager position she still gets support from the organisation leadership and organisational HR policies to be able to advance. ”. IDI_ mid-level manager_ Female_ Private*

Other female senior managers in the private sector, reported that women were prioritized during promotion because the projects the institutions were implementing focused on girls who would only be comfortable engaging with people of the same gender.

*“Most projects are headed by women…..because we give preference during promotion to women since we mostly deal with girls and they are more comfortable with their gender”.KII_senior-level manager_Female_Private*

Regarding the public sector, some managers noted that gender equity is sometimes considered during promotion in addition to academic and professional qualifications to ensure that there is a gender balance during promotion.

#### b) Flexible work schedules

Flexible work hours were thought by some female middle-level managers in the private sector to have contributed to women’s promotions to leadership positions given that women who are enrolled in school are allowed to leave work early to pursue their studies and after completion of these studies they have risen in leadership ranks. For example, they mentioned that a woman who had a certificate at the point of employment was able to advance her studies and is currently pursuing a Master’s degree because of the flexible working hours. This advancement in her education qualification has contributed to her career advancement.

*“Maybe I can give an example, because there is a policy here that allows people to advance their career, if you came here with an acceptance letter from university or a school that says you need to be going to evening classes. You submit that to the HR and at 3 [pm] you are let to go. So and it is open to everybody, so this particular woman took that. Started certificate level, went to diploma, went to degree now is doing masters finishing. She is doing very well; she has risen also”. IDI_ mid-level manager_ Female_ Private*

The excerpts from the interviews with the health managers did not distinctively show that policies and practices relating to hiring, equal pay, parental and care leave, reproductive rights, and sexual harassment had a role in women’s career advancement. However, they implied that these policies and practices contribute to inclusivity during the hiring and retention of women in the health sector workforce. The perspectives of the managers on these five sub-themes based on their sex and sector are described below.

#### c) Hiring

Male HR managers in the public sector had different opinions on whether gender is a consideration during their hiring process. While some of them mentioned that competence and meeting the qualifications are the key considerations during the hiring process, others reported that they deliberately engage in positive discrimination whereby they ensure that gender is considered during recruitment so that more women can be hired for gender balance. This is guided by the constitution's two-thirds gender rule which advocates for having a gender balance during appointments in public offices by ensuring that not more than two-thirds should be of the same gender [7].

*“We follow the Constitution’s two-thirds gender when we do something like recruitment .We are trying to mainstream the gender. We are diverse to have a gender balance, so that we bring women on board”. KII_senior-level manager_Male_Public*

#### d) Equal pay

Most female and male senior-level managers in both the public and private sectors reported that pay is not determined by sex but rather by one’s education qualification, work experience, competence and organisation’s budget. Notably, some female managers mentioned that they did not know their colleagues’ salaries, but thought that they were paid almost the same as their male counterparts.

*“I don’t know much about equal pay because I don’t know about salaries but our male colleagues... I think we are paid almost the same. Here is just papers and your experience, through your appraisals, promotions, like things are done that way”. IDI_ mid-level manager_ Female_ Public*

#### e) Sexual harassment

Both female and male senior-level and middle-level managers from the public and private sectors reported that they have safeguarding policies focusing on sexual harassment and gender-based violence at the workplace, either as a standalone policy or embedded in the HR manual. Sexual harassment policies guide how colleagues interact with other each in the workplace.

*“We have a sexual exploitation and harassment policy…This guides on addressing your colleagues in a respectful way and how we also treat each other in the office”. IDI_ mid-level manager_ Female_ Private*

#### f) Parental leave

The majority of senior managers stated that there are policies in place that enable women to take paid maternity leave for a certain amount of time before returning to work. However, the duration of the leave varies for different institutions in the public and private sectors. Most male and female senior-level and middle-level managers in the public sector mentioned that maternity leave was 90 days given that they follow the government’s directive. On the other hand, for the private sector, one female HR manager noted that staff get 30 days maternity leave.

*“Policies relating to reproductive health rights for employees are also embedded in our HR manual …where you get your thirty (30) days maternity paid leave”. KII_senior-level manager_Female_Private*

Regarding paternity leave, only a few middle-level managers in both public and private sectors indicated that there is provision for this type of leave within their policies. The duration of the paternity leave was not specified for the majority of institutions. However, for those that specified, the duration of the paternity leave varies between 7 to 10 days.

#### g) Reproductive rights

Some female health managers in the private sector reported that women employees in their institution can access reproductive healthcare services because their organisation provides insurance benefits that cover their reproductive health needs. On the other hand, some male and female health managers in public sector institutions noted that either they have provisions for a small room for their female employees to express and store milk or dedicated crèche facilities for the staff’s young children.

*“Then there are things like those mothers who are breastfeeding, we have a breastfeeding corner here which is meant for staff manned by a nutritionist. So we encourage our staff that once you have gone for leave which is usually 90 days’ maternity leave, if it is over, you are supposed to breastfeed for 6 months. So you are free to come with your baby, leave the baby at the breastfeeding corner, come work at a particular time go breastfeed come back”. ”KII_senior-level manager_Male_Public*

Some private and public sector female and male managers pointed out that they have policies that allow flexibility for women who have delivered or breastfeeding to leave work early or to have flexi hours.

*“One when you are expecting, especially after delivery we leave work at 3.00pm as a mother for 6 months”. IDI_ mid-level manager_ Female_ Public*

### Implementation and monitoring of health sector workplace policies and practices

The health managers from both private and public sectors only reported how all the policies and practices within their institutions are implemented and monitored in general; however, they did not state how the specific policies for example a policy on promotion is being implemented or monitored. While most of these managers mentioned that the implementation and monitoring process of all the workplace policies exists within their institutions, few others cited the lack of formal structures allowing the proper transition of personnel tasked with implementation and monitoring making it difficult for this process to take place.

*“We are so informal. The board should do the monitoring and are supposed to be looking into different issues pertaining to the employees. We always have them (board) after every three years, after their term is over…So every time we postpone issues from one board to another and terms end before these issues are looked into”. IDI _ mid-level manager _ Female _ Public*

Health managers in the health public sector organisations noted that policies within their institutions are implemented and monitored centrally at the county level by the Department of Health (for devolved public sector organisations such as hospitals) or at the national level by the Ministry of Health for non-devolved public sector organisations such as medical councils. On the other hand, their counterparts in private sector organisations stated that their policies are implemented and monitored at the individual institution level by the Management Board and HR.

*“That one is also from the ministry (of health). They are the ones who come up with formulation of policies, they are the ones who do the monitoring and evaluation, so for us we don’t do that”. KII_senior-level manager_Male_Public*

Regardless of whether the implementation and monitoring of policies is done at the county, national or institutional level, both senior and middle-level managers in different sectors reported that different units or people have been tasked with this role. Some respondents both in the private and public sector indicated that it was the duty of the HR department to implement the policies while others mentioned that this is the role of management/leadership. Notably, for some public sector organisations, this role was reported to be bestowed to a gender committee comprised of members with different seniority levels whose main focus is to handle issues and policies dealing the gender issues as well as gender mainstreaming in general.

*“Implementation of the policies is under the HR department. They are coming up with some documents and we hope to adopt them soon, under their monitor”. IDI _ mid-level manager _ Female _ Public*

*“Yes, they (Gender committee) come up with the implementation. You know for ease of operation and work you delegate”. IDI _ mid-level manager _ Male _ Public*

According to some health managers within the private sector, their policies begin to be implemented and monitored during the hiring process as employees are given policies to append their signatures to hold them accountable, others in the same sector, noted that their leadership conducts periodic dissemination of the policies as a way of keeping their staff informed about the policies in place.

*“When you formulate policies you have to disseminate them. When we have our Mid-Management meetings like early in the year in February, they are three in a year. Policies are disseminated to the head of the departments or project and they disseminate to the lower cadre. If there is anything new or any change, it is always disseminated to staff members”. KII_senior-level manager_Female_Private*

To ensure effective implementation and monitoring of policies, a female middle-level manager in the private sector reported that her institution conducts regular checks to ensure that staff adhere to the policies in place and are updated on any changes that have happened in the respective policies.

*“MD office and HR does regular check-ups; checking people's files if they have signed these policies, if they are adhering to them and they keep asking if there are any questions. If there are updates that is given to everyone”. IDI _ mid-level manager _ Female _ Private*

On the other hand, a male middle-level manager in the public sector said that his organisation benchmarks with other institutions to determine what works for them. They do this by visiting other institutions to see what policies on gender they have and how they implement them. Using this experience, they then develop similar policies and practices.

Failure to adhere to the policies in place, both male and female middle-level managers in the private sector noted that there are consequences such as loss of jobs.

“*Breach of, especially something like the child protection policy, sexual harassment policy, whistleblowing…if something is raised, might lead to someone losing their jobs”. IDI _ mid-level manager _ Female _ Private*

## Discussion

In this study, we examined the knowledge and perspectives of health managers in Kenya on their organisations' workplace policies and practices contributing to the career advancement of women. We also examined their knowledge and perspectives on which of their organisations’ policies and practices contribute to the career progression of women and their knowledge of how such policies and practices are implemented and monitored. The study findings indicate health sector managers especially middle-level managers in both the private and public sectors in Kenya have limited awareness of the policies and practices that can advance women’s career progression in their organisations. This could be attributed to either a lack of proper orientation on the policies and practices or limited policies and practices within the institutions that can contribute to women’s career advancement altogether. This finding shows the need for institutions to offer continuous information sessions to their staff on the policies and practices that exist to advance women’s careers.

We also found out that these organisations have limited policies and practices that can advance women’s career progression. The only policies in place as reported by a few health managers are related to promotion and flexible work schedules. This shows the need for organisations in this sector to be deliberate in having supportive policies as well as other pragmatic interventions beyond these policies such as training and mentorship that can enable women to progress in their careers. This is because existing evidence [3, 11, 14] has shown that policies are important, but not sufficient to create inclusive environments for women to advance in their careers. The policies should be combined with capacity-strengthening or building initiatives such as training on various skills, professional mentorship, and awarding education scholarships as well as having a conducive organisation culture in which they can thrive. The social-cultural norms that perpetuate gender-based discrimination and inequality in the workplace should also be addressed [1, 3].

One notable finding from the interviews with the health managers is that women’s promotion is considered appropriate most often when running women-focused programs but not when running programs that do not focus on women or girls. This portrays a lack of intentionality by some organisations to have women leaders excerpt when prompted by circumstances based on the project design and thematic area. Organisations need to appoint women into leadership positions regardless of whether the programs are women or men-focused as long as they have the required skillsets to deliver the tasks related to the programs. Notably, the excerpts from the interviews attribute a woman’s promotion to a leadership position for the first time to the newly formulated organisational policies that encourage women’s career progression. Our finding is consistent with a systematic review by Mousa et al. [14] which illustrates that for organisations to achieve transformational shifts in leadership, effective policies are mandatory.

While the Constitution of Kenya, 2010 has a provision to ensure that there is gender balance during appointments [7], our study findings illustrate how some public sector organisations are not actively addressing gender bias and discrimination during the hiring process. While interviewees state that they focus on candidate qualifications and not gender, evidence shows how gender bias can negatively impact women’s opportunities at each step in the recruitment and hiring process [3, 12, 15–17]. To achieve gender balance, affirmative action needs to be enforced by organisations as some of the organisations in this study were doing such intentionally prioritizing recruiting women in the event that there are relatively fewer female employees than male employees. This will also ensure that more women can join the health-sector workforce. Moreover, this approach contributes to the ongoing efforts to redress the historical imbalance that has marginalized women in Kenya like other countries in the region [17].

A key finding from this study is that for both private and public sector organisations, pay is not determined by gender but by other factors such as education qualification, work experience, competence, and the organisation’s budget. This finding differs from other studies which indicate that pay gap still exists in many settings and that is mainly driven by many women working in low-paying jobs as compared to high-paying jobs [2, 4, 5]. While our study respondents did not state that there are pay differences because of gender, does not mean that the issue may not exist given that there is still supporting evidence to show it still exists. Moreover, none of the study participants mentioned if equal pay is assessed or audited and hence it is difficult to determine whether equal pay exists in these institutions as claimed. There is a need for further mixed-methods research in Kenya to interrogate this finding.

Policy related to sexual harassment were missing in some institutions and yet more than 70% of women are reported to have experienced sexual harassment in a professional capacity [18]. Sexual harassment has a devastating impact on women workers’ health, wellbeing and performance. This limits their ability to compete fairly with their male counterparts. It is also a major barrier to achieving equality of opportunity and access to decent and dignified work [18]. There is a need for all organisations to have a sexual harassment policy so as to create an enabling environment for women to thrive in workplaces.

Paid maternity leave contributes to more women remaining in the workforce [3] and improves employees' morale and the health and wellbeing of their families [19]. However, the duration of the paid maternity leave might pose as a challenge to women continuing to work. While Article 29(3) [20] of the Employment Act provides for up to 90 days of maternity leave, our study shows that this law is mostly followed by public sector organisations. Our study suggests that some private sector organisations do not abide by this law. We found that some only provide 30 days maternity leave. Given this short maternity leave, some women may likely leave work and take care of the infants. This in turn affects the woman economically. This finding suggests the need for the government to ensure that maternity leaves are streamlined across all organisations in accordance with the law.

While our study reveals that a good number of public and private sector organisations have mechanisms in place to implement and monitor the implementation of all policies in general, it also highlights the issue of lack of knowledge of the health managers on how the specific policies for example policies on promotion are implemented and monitored. General knowledge of how the policies are implemented and monitored is salient, however, having awareness of mechanisms to which specific policies are implemented and monitored is equally important for example if the employees have grievances that relate to specific policies. In addition, there is a need for transparency in reporting on policies and outcomes by management such as gender pay gap or staff distribution so that the staff are informed if policies are being implemented and whether they are effective. This study also highlights the issue of policies being implemented and monitored at the county or national level for the majority of public sector organisations. This has contributed to some managers not fully aware of the policies. While having a centralized system of handling policies for public sector organisations may be important in managing many public health sector organisations, this also poses as a challenge given that different institutions in the public health sector have unique needs which may not be properly captured if one document is used across the board. While streamlining is important within the sector, it is also important to recognize individual institution’s needs and customize policies to that effect. Our study suggests that each public-sector organisations should have their own workplace policies governing their day to day operations.

The findings of this study suggest that to have more women join, be retained, and thrive in the health sector workforce, both public and private sector organisations need to be deliberate in having effective policies and practices that create an enabling environment for them. In addition, these policies and practices should be developed in line with the laws in the country especially for private sector organisations so that no women are disadvantaged. Moreover, implementation and monitoring of policies for public sector organisations need to be conducted at the institutional level so that the unique needs of the individual institutions are addressed. Organisations should have continuous information sessions with their staff on the policies and practices that exist to advance women’s careers and be transparent in their implementation and monitoring for accountability.

### Study limitations

Given that workforce policies and gender are sensitive issues, there is a high likelihood that our study could suffer from reporting bias, especially among human resources managers. We tried to mitigate this limitation by conducting the interviews in private spaces to engender trust and confidence among respondents, thereby ensuring the accuracy of reporting. The health sector is broad and it includes organisations that range from research institutions pharmaceutical companies, regulatory bodies, etc. thus it is impossible for all these categories to be represented in a qualitative study. However, we tried to ensure that the opinions shared would be cross-cutting by phrasing the questions in a broader way. While our study is among the few studies providing evidence on organisations’ workplace policies' contribution to the career advancement of women, we only provide the data on what workplace policies and practices contribute to women’s career progression and not how these policies do so because this was an exploratory study. Future research may focus on how these workplace policies contribute to women’s career progression to elicit more insights in this area.

## Conclusion

This study demonstrated that health sector managers in both the private and public sectors in Kenya have limited awareness of policies and practices that can advance women’s career progression. This could be attributed to either a lack of proper orientation on the policies and practices or limited policies and practices within the institutions that can contribute to women’s career advancement altogether. This shows the need for institutions to offer continuous information sessions to their staff on the policies and practices that exist to advance women’s careers and be transparent in their implementation and monitoring. In addition, there is a need for organisations in this sector to be deliberate in having supportive policies as well as other pragmatic interventions beyond policies such as training and mentorship that can enable women to progress in their careers. Our study also illustrated the need for each public sector organisation to develop, implement and monitor their own policies to increase ownership of the policies and to address the distinctive needs of the institutions.

## Data Availability

The qualitative data analyzed during the current study is currently not publicly available as it is waiting to be published on the institution&apos;s microdata portal after documentation. However, it can be shared on reasonable request to the corresponding author.
